# Effects of atorvastatin on chronic subdural hematoma: A systematic review: Erratum

**DOI:** 10.1097/MD.0000000000007616

**Published:** 2017-07-21

In the article, “Effects of atorvastatin on chronic subdural hematoma: A systematic review”,^[1]^ which appeared in Volume 96, Issue 26 of *Medicine*, Dr. Liang Shen's affiliation should have been ^a^ Department of Neurosurgery, Huzhou Central Hospital, Huzhou, Zhejiang.
